# NF-κB in Hematological Malignancies

**DOI:** 10.3390/biomedicines5020027

**Published:** 2017-05-31

**Authors:** Véronique Imbert, Jean-François Peyron

**Affiliations:** Centre Méditerranéen de Médecine Moléculaire, INSERM U1065, Université Côte d’Azur, 06204 Nice, France; Jean-Francois.PEYRON@unice.fr

**Keywords:** NF-κB, leukemia, lymphoma

## Abstract

NF-κB (Nuclear Factor Κ-light-chain-enhancer of activated B cells) transcription factors are critical regulators of immunity, stress response, apoptosis, and differentiation. Molecular defects promoting the constitutive activation of canonical and non-canonical NF-κB signaling pathways contribute to many diseases, including cancer, diabetes, chronic inflammation, and autoimmunity. In the present review, we focus our attention on the mechanisms of NF-κB deregulation in hematological malignancies. Key positive regulators of NF-κB signaling can act as oncogenes that are often prone to chromosomal translocation, amplifications, or activating mutations. Negative regulators of NF-κB have tumor suppressor functions, and are frequently inactivated either by genomic deletions or point mutations. NF-κB activation in tumoral cells is also driven by the microenvironment or chronic signaling that does not rely on genetic alterations.

## 1. Introduction

The NF-κB family of transcription factors coordinates inflammatory responses, innate and adaptive immunity, cellular differentiation, proliferation, and survival in all multicellular organisms.

The NF-κB system is tightly controlled at various levels, and deregulations of NF-κB homeostasis have been implicated in a wide range of diseases, ranging from inflammatory and immune disorders to cancer [1,2]. In particular, NF-κB is a key link between chronic inflammation and cancer transformation [3].

The mammalian NF-κB family is composed of five members: RelA (p65), RelB, c-Rel, NFKB1 (p105/p50), and NFKB2 (p100/p52), which form various dimeric complexes that transactivate numerous target genes via binding to κB consensus DNA binding sites. They are tightly regulated by the IκBs protein family including typical IκBs (IκBα, IκBβ, IκBε), the precursor proteins p100 and p105, and the atypical IκBs (IκBζ, BCL-3, IκBη). The typical IκBs and the precursors are expressed in the cytoplasm to sequester NF-κB dimers by masking their nuclear localization sequence. On the contrary, the atypical IκBs are hardly expressed in resting cells, and are induced upon cell activation to interact with NF-κB dimers within the nucleus. They can act as activators or inhibitors of NF-κB, depending on which proteins of the transcriptional machinery they recruit.

NF-κB activation is mediated by two signaling pathways as detailed in [4,5,6].

The canonical or classical pathway—the most widely known route to NF-κB activation—is essentially mediated by the action of the RelA/p50 subunits. In resting conditions, RelA/p50 dimers are retained in the cytoplasm complexed with the IκBα inhibitor. Upon cell activation by pro-inflammatory cytokines, immune receptors engagement, and stress conditions, IκBα is first phosphorylated on two serine residues (Ser32 and 36 in human IκBα) by the IκB-kinase (IKK) complex, which is composed of two kinases—IKKα (or IKK1) and IKKβ (or IKK2)—associated with an essential scaffold protein IKKγ (or NEMO, NF-κB essential modulator). The activation of the IKK complex depends on extracellular signals: hence, T- and B-cell antigen receptors (TcR and BcR) are coupled to the CARMA/BCL10/MALT1 (CBM) complex to activate IKKs, whereas the receptors for IL1 (Interleukin 1), TNFα (Tumor Necrosis Factor-α), and toll-like receptors (TLRs) rather engage a signaling complex involving TRAFs (TNF Receptor Associated Factor), IRAKs (interleukin 1 receptor associated kinase 1), LUBAC (Linear UBiquitin Assembly Complex), and TAK1 (TGF-β Activated Kkinase 1) proteins. IκBα phosphorylation triggers the recruitment of the E3 ubiquitin ligase SCF/βTRCP (Skp, Cullin, F-box/transducing repeat containing protein) and the subsequent polyubiquitination of IκBα, marking it for degradation via the 26S proteasome. This degradation releases the RelA/p50 dimer that can translocate into the nucleus to activate the transcription of target genes.

The non-canonical or alternative pathway activates mainly RelB-p52 complexes through the inducible processing of p100. In contrast to the canonical pathway, this pathway is activated by a more restricted number of ligands, such as the B-cell-activating factor (BAFF) belonging to the TNF family, CD40L, lymphotoxin β (LTβ), receptor activator nuclear factor ligand (RANKL), or CD30L. The triggering of these cell surface molecules engages the assembly of a signaling complex that involves cellular inhibitor of apoptosis (cIAP1 and cIAP2), TRAF2, and TRAF3. Receptor engagement leads to the recruitment and activation of cIAP1/2 mediated by TRAF2, resulting in the degradation of TRAF3, stopping the continuous degradation of the NF-κB-inducing kinase (NIK) that is central to this pathway. NIK can then activate IKKα, which phosphorylates p100. This provides a signal for their recognition by the SCF/βTRCP ubiquitin ligase complex and the proteasome-mediated processing of p100 by in situ degradation of the IκB-like domain of the precursor proteins, producing mature p52 protein. The mature RelB/p52 dimers are then released, translocate into the nucleus to start the transcription of their target genes. NF-κB activation strongly relies on multiple connected biochemical reactions—in particular on three main types of ubiquitination events to build (K63, linear) or disassemble (K48) signaling complexes (see [7] for a review).

**Figure 1:**
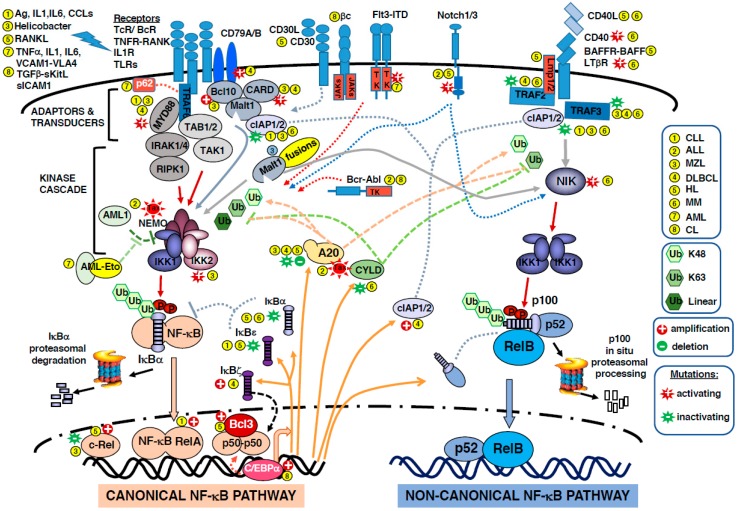
Deregulations of NF-κB in hematopoietic malignancies. The figure depicts the different actors in the canonical and non-canonical NF-κB activation pathways and their involvement in leukemia and lymphoma. The disease in which a given protein is involved is identified by a code number in a yellow filled circle 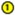. 1- CLL: chronic lymphocytic leukemia; 2- ALL: acute lymphoblastic leukemia; 3-MZL: marginal zone lymphomas; 4- DLBCL: diffuse large B-cell lymphomas; 5- HL: Hodgkin lymphoma; 6- MM: multiple myeloma; 7- AML: acute myeloid leukemia; 8- CML: chronic myeloid leukemia. The participation of ubiquitination reactions in signaling is not detailed but highlighted with a green color code; K48 ubiquitination is responsible for degradation, K63 and linear are involved in the assembly of signaling complexes. Gene amplification/deletion are indicated by 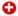 and 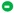, respectively. Mutations affecting the genes for NF-κB subunits or actors of the NF-κB pathways are mentioned as activating 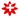 or inactivating 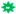 mutations. 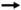 illustrates positive action whereas 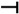 illustrates inhibitory action. Color code: color of the arrow refers to the protein concerned by the depicted action. Plain orange arrow shows the proteins of interest after transcription via NF-κB. Plain red arrow shows phosphorylation events. Dotted red arrow shows tyrosine phosphorylation. Abbreviations used are: Ag: antigen; IL: interleukin; CCL: C-C motif chemokine ligand; RANKL: receptor activator of NF-κB ligand; TNF: Tumor Necrosis Factor; VCAM1: vascular cell adhesion molecule 1; VLA-4: very late antigen-4; TGF: transforming growth factor; KitL: kit ligand; ICAM1: intercellular adhesion molecule1; TcR/BcR: T-cell or B-cell antigen receptors; TNFR: TNF receptor; TLR: toll-like receptor; CD: cluster of differentiation; βc: common β chain of interleukin receptors; JAK: Janus kinase; TK: tyrosine kinase; Flt3-ITD: colony-stimulating factor receptor 1-like-3-internat tandem repeat; Notch: notch receptor; BAFF: B-cell activating factor; LTαR: lymphotoxin α receptor; LMP1: latent membrane protein; TRAF: TNF receptor associated factor; c-IAP: cellular-inhibitor of apoptosis; p62 = SQSTM1: sequestosome 1; Myd88: myeloid differentiation primary response gene 88; Bcl10: B-cell leukemia/lymphoma 10; CARD: protein with a caspase recruitment domain; Malt1: mucosa-associated lymphoid tissue lymphoma-1; TAB: TAK1 binding protein; TAK1: TGF-β activated kinase 1; IRAK: interleukin 1 receptor associated kinase 1; RIPK1: receptor (TNFR)-interacting serine-threonine kinase 1; Bcr-Abl: breakpoint cluster region-abelson kinase, product of the t(9; 22) translocation; NIK: NF-κB-inducing kinase; AML1: acute myeloid leukemia gene 1 = RUNX: Runt-related transcription factor 1; AML-Eto: product of the t(8; 21) translocation; Tax: HTLV-1 viral protein; IKK: inhibitor of κ-B kinase; NEMO: NF-κB essential modulator/inhibitor of NF-κB subunit γ; A20 = TNFAIP3: TNF α-induced protein 3; CYLD: cylindromatosis gene product lysine 63 deubiquitinase; IκB: inhibitor of NF-κB; Rel: reticuloendotheliosis proto-oncogene, NF-κB subunit; C/EBP: CCAAT/enhancer binding protein.

The two pathways are interconnected, as the canonical one regulates p100 and RelB levels [8]. Both canonical and non-canonical NF-κB activation pathways have been implicated in human hematological malignancies, mainly lymphoid leukemia and lymphoma. Because NF-κB regulates a large array of target genes, the constitutive activation of NF-κB can support most steps involved in cancer transformation: inhibition of cell differentiation and apoptosis, promotion of cell proliferation, angiogenesis, cancer-related inflammation and metastatic potential, and resistance to treatments. The constitutive activation may have different origins. It can result from rearrangements and mutations in genes encoding NF-κB or IκB members, or in genes encoding upstream components of the cascade, but it may also derive from persistent autocrine or paracrine signaling and/or hyper-activation of immune receptors (TcR, BcR, TLRs). In this review article, we present the role of the NF-κB pathway and subunits in human hematologic malignancies. Figure 1 summarizes the molecular partners involved in the two pathways to NF-κB activation, as well as the dysregulations that occur in hematologic malignancies. For a broader description of the role of NF-κB subunits in cancer, see [9].

## 2. Lymphoid Malignancies

### 2.1. Leukemias

Chronic lymphocytic leukemia (CLL) is characterized by the progressive accumulation of mature monoclonal B lymphocytes in the peripheral blood (PB), bone marrow (BM), and secondary lymphoid organs such as lymph nodes (LN). NF-κB is constitutively activated in CLL patients [10,11]. The microenvironment exerts a critical role in the natural history of CLL. Indeed, signals from multiple receptors (BcR, TLR, CD40) result in the activation of downstream pathways, including NF-κB [12,13]. In addition, there is evidence that the NF-κB pathway and its upstream mediators can be targeted by recurrent genetic lesions in some minor cases. For example, 3% of CLL patients display the L265P mutation on MYD88 (MYeloid Differentiation primary response gene 88). This mutation changes the structure of MYD88 to allow spontaneous homodimerization and recruitment of the serine/threonine kinases IRAK1 and IRAK4 that are essential for NF-κB activation by the TLRs. This enhances the responses to TLR ligands, leading to a higher release of cytokines (IL6—Interleukin 6—, IL1RA—Interleukin 1 Receptor Agonist—) and chemokines (CCL2/CCL3/CCL4 —Chemokine Ligand 2/3/4—). These cytokines/chemokines have been reported to be important in attracting other cell types (e.g., T lymphocytes) by CLL cells to create an advantageous microenvironment supporting leukemic survival [14]. The most frequently mutated gene (7% of CLL cases) in CLL within the NF-κB pathway is *NFKBIE* that encodes IκBε—a negative NF-κB regulator. *NFKBIE* aberrations were highly enriched in poor-prognostic subgroups, demonstrating the supporting role of NF-κB for transformation. These aberrations lead to reduced IκBε protein levels, diminished interactions with RelA, as well as increased phosphorylated RelA and nuclear translocation [15]. *BIRC3—Baculoviral IAP repeat–containing protein 3—*(the gene encoding for the cIAP2 ubiquitin ligase) is also mutated in CLL. cIAP2, along with TRAF2 and TRAF3, cooperates in the same protein complex to negatively regulate NIK, the central activator of non-canonical NF-κB signaling. All inactivating *BIRC3* mutations detected in CLL are predicted to cause the elimination or truncation of the C-terminal RING domain, the E3 ubiquitin ligase activity which is essential for NIK proteasomal degradation. The mutations impair NIK ubiquitination, and thereby favor its stabilization, leading to the phosphorylation of NFKB2 and the processing of p100 to p52. This results in constitutive NF-κB activation, as evidenced by the detection of a higher p52/p100 ratio in *BIRC3*-mutated patients [16,17]. If *BIRC3* mutations are rare in early stages of CLL, they tend to accumulate as the disease progresses, suggesting a selective advantage for the transformed cells bearing this mutation. Finally, analysis of a cohort of 131 CLL patients revealed that DNA binding of RelA is constitutively elevated in patients with more aggressive disease, and is also further induced by conventional chemotherapy, which in turn seems to contribute to the depth of response to subsequent treatment cycles. That study identified RelA as a superior prognostic marker for the survival of CLL patients, and crucially demonstrates that RelA levels have the potential to predict the duration of the response to therapy [18].

Some epigenetic changes and aberrant microRNA expression have also been associated with NF-κB dysregulation in CLL. For example, the silencing of miR-9-3a tumor suppressor miRNA by methylation may account for the constitutive upregulation of *NFKB1*, and hence the constitutive activation of NF-κB in CLL patients [19]. In addition, the miR-708 enhancer is aberrantly methylated in CLL. miR708 directly targets IKKβ, and thereby leads to the repression of NF-κB signaling. CLL patients with high methylation of the miR708 enhancer present a poor prognosis [20].

Acute lymphoblastic leukemia (ALL) is mainly a disease of childhood that arises from recurrent genetic insults that block the differentiation of B- and T-cell precursors to drive their aberrant cell proliferation and survival. ALL subgroups with distinct biological characteristics are frequently characterized by dysregulated transcription factors or kinases—most prominently the fusion proteins TEL-AML1 (Translocation Ets Leukemia-Acute Myeloid Leukemia 1 protein) encoded by t(12;21)(p13;q22) in presumed good risk ALL, or Bcr-Abl (Breakpoint cluster region-Abelson murine leukemia virus) encoded by t(9;22)(q34;q11) and MLL-AF4 (Mixed Lineage Leukemia-ALL1 Fused gene on chromosome 4) by t(4;11)(q21;q23) in high risk ALL. ALL in adults is characterized by a higher frequency of high-risk cytogenetics and a lower incidence of favorable genetic abnormalities.

The vast majority of ALL patients present a constitutive activation of the canonical NF-κB pathway in the form of RelA/p50 complexes, which is an important switch to ensure the survival of ALL cells by blocking apoptosis or enhancing cell proliferation [21].

T-cell acute lymphoblastic leukemia (T-ALL) is an aggressive malignancy of transformed thymocytes that mainly affects children and adolescents. Although mutations in *NF-κB* genes have not been reported in T-ALL (unlike other lymphoid malignancies), constitutive activation of NF-κB frequently occurs in primary human T-ALL and T-ALL mouse models. Kordes and colleagues showed by electrophoretic mobility shift assays [21] that at diagnosis, childhood T-ALL cells (11 of 13 cases) displayed a constitutive activity of NF-κB consisting of RelA-p50 dimers associated with IκBα phosphorylation. Another study realized on human T-ALL cell lines depicted a constitutive NF-κB activity associated with constitutive IKK activity and nuclear localization of all the NF-κB members (p50, p105, RelA, RelB, and c-Rel), suggesting the activation of both canonical and non-canonical pathways. Somatic activating NOTCH1 mutations are found in more than 50% of human T-ALL cases, and they result in elevated levels of ICN1—the transcriptionally-active intracellular domain of NOTCH1. NOTCH1 can mobilize both the NF-κB canonical signaling by activating the IKKα/β/γ complex and the non-canonical pathway by inducing the expression of RelB and NFKB2 and activating IKKα homodimers [22]. The mechanism of Notch-induced NF-κB activation in T-ALL involves Hes1, which transcriptionally represses CYLD (cylindromatosis), a deubiquitinase which down-regulates NF-κB signaling by removing the activator K63 ubiquitin chains from different elements of the NF-κB signalosome [23]. In transgenic mice, NOTCH3 triggers both classical and alternative NF-κB activation pathways, depending on the expression of the pre-TcR. Indeed, in the absence of pre-Tα/pre-TcR, Notch 3 induces IKKα, inducing a higher p100 substrate processing and a release of RelB/p52 complexes [24]. Bcr-Abl expression in either T-ALL or B-ALL cells triggers an IKK-dependent activation of NF-κB that is crucial to the pathogenicity of Philadelphia positive (Ph+) leukemias [25].

Chronic inflammation directed by NF-κB is vital for the pathogenesis of adult T-cell leukemia (ATL)—an aggressive malignancy with a poor prognosis that is induced by the human T-cell leukemia virus type I. HTLV-1 encodes a 40 kDa oncoprotein (Tax) that regulates viral gene expression and plays vital roles in ATL leukemogenesis. Tax interactions with Ubc13 (E2-conjugating enzyme), NEMO, TAX_1_BP_1_ (Tax Binding Protein), and NRP (NEMO related Protein) are critical for activation of the IKK complex [26,27,28]. Tax also maintains persistent NF-κB activation by inactivating NF-κB negative regulators such as TNF α-induced protein 3 (TNFAIP3) and CYLD [29].

Finally, activation of NF-κB was identified as a mechanism for resistance to IFNβ in the poor-prognosis MLL-ALL subtype (t(4;11) translocation) [30].

### 2.2. Lymphomas

Marginal zone lymphomas (MZLs) are indolent small B-cell lymphomas classified in three subgroups, depending on the localization: mucosa-associated lymphoid tissue (MALT), splenic (sMZL) and nodal (nMZL) lymphomas. Both canonical and non-canonical NF-κB pathways can be activated through a variety of mechanisms, from molecular alterations to epigenetic modifications—especially in MALT and splenic MZL lymphomas.

In fact, MALT lymphomas represent an archetypal example of the link between chronic inflammation and tumor development. In particular, gastric MALT lymphomas develop from a background of chronic gastric infection with *Helicobater pylori*, while those from the skin and ocular adnexa or diseases of the small intestine are either associated with chronic infection with *Borrelia burgdorferi*, *Chlamydia psittaci*, or *Campylobacter jejuni*. The prolonged chronic microbial infection generates immune and inflammatory responses that transform the polyclonal B-cell population into a monoclonal B-cell lymphoma. At the early stage of MALT lymphoma, eradication of *H. pylori* infection by antibiotics causes tumor regression [31]. Thereafter, acquisition of chromosomal translocations constitutively activating NF-κB provides antigenic independence and antibiotic resistance. The t(11;18)(q21;q21) translocation represents the most frequent modification in 18% of MALT lymphomas, and can be enriched to 40% in gastric MALT lymphomas. It generates an cIAP2-MALT1 fusion which oligomerises, triggering TRAF6-dependent ubiquitination of NEMO, leading to the activation of the canonical NF-κB pathway [32]. In addition, the auto-oligomerization of cIAP2-MALT1 also induces the recruitment of NIK and its subsequent cleavage by the MALT1 protease domain, leading to degradation-resistant NIK kinase and deregulated non-canonical NF-κB signaling [33]. The t(14;18)(q32;q21) chromosomal translocation is very frequent in MALT lymphoma (10–20%), but is never found in the gastric forms. This translocation leads to the IGH–MALT1 fusion and causes MALT-1 overexpression, and thereby enhances the canonical NF-κB signaling [34]. The t(1;14) (p22;q32) translocation which juxtaposes the *BCL10* (B cell lymphoma/leukemia 10) gene under the regulatory control of the Ig heavy chain gene enhancer (B*CL10-IGH*) is a rare genetic aberration. Over-expression of BCL10 causes its constitutive activation through oligomerization via its N-terminal CARD/CARD (Caspase Activation and Recruitment Domain) interaction, and thus leads to enhanced NF-κB activity [35]. Intriguingly, BCL10 protein is aberrantly expressed in the nuclei of lymphoma cells, suggesting an as-of-yet unappreciated role of nuclear BCL10 in the pathogenesis of MALT lymphoma [35].

An aberrant NF-κB activation is found in 30–40% of splenic MZL patients. This involves dysregulation of the canonical pathways due to molecular lesions in a number of genes belonging to the NF-κB pathway. First, inactivation of the negative regulator *TNFAIP3* (A20) by non-sense or frame-shift mutations is found in 10–15% of patients [36]. TNFAIP3 is responsible for switching off signals converging from surface receptors on NF-κB as well as the inhibition of NF-κB proteins. Consequently, *TNFAIP3* disruption in MZL causes supraphysiological activation of NF-κB signaling. Two activating mutations (K171E and K171T) affecting IKKβ were found in 10% of splenic MZL [37,38]. The L265P mutation of MYD88—which affects 3% of MZL patients—changes the structure of MYD88 to allow spontaneous homodimerisation and recruitment of serine threonine kinases IRAK1 and IRAK4 essential for NF-κB activation by the toll-like receptors [39]. In addition, CARD11 coiled-coil domain mutations (5–10% of patients) promote spontaneous CARD11 multimerisation and association with other components of the CBM complex, thus leading to IKKβ kinase activation and NF-κB upregulation [39]. In some splenic MZL patients, activation of the non-canonical NF-κB pathway has also been described. BIRC3 RING domains mutations are found in 10–15% of patients. As detailed above, these mutations affect NF-κB activation by stabilizing NIK. Finally, in 5% of MZL patients, TRAF3 is inactivated by mutations causing elimination of the C-terminal domain of the protein. This domain is involved in the docking of NIK and its recruitment to BIRC3 degradation. So, TRAF3 mutations also stabilize NIK to upregulate non-canonical NF-κB signaling [39].

Rare mutations (0.3%) affecting an IKK phosphorylation site on c-Rel’s transactivation domain have been described in two B-cell lymphomas [40]. Cells expressing this mutation appeared less sensitive to TNF-α-induced apoptosis, and consequently may be more prone to transformation by other oncogenic events affecting these cells.

Diffuse large B-cell lymphomas (DLBCL) are the most common types of non-Hodgkin lymphoma (40% all adult cases). They are divided into three molecular sub-types: ABC (activated B-cell) GCB (germinal center B-cell) and PMBL (primary mediastinal B-cell lymphoma). Initial evidence for the role of the canonical NF-κB pathway in DLBCL came from gene expression profiling studies that revealed a significant enrichment for NF-κB target genes specifically in the ABC subgroup, which display the worst prognosis [41]. This was then confirmed by the detection of RelA/p50 complexes in the nuclei of DLBCL tumors [42]. Constitutive NF-κB activation in ABC-DLBCL can result from mutations in components of the BcR signaling cascade, as ABC-DLBCL cells exhibit a chronic BCR activation. It is sustained by mutations in the two chains of the CD79 complex which forms the B-cell receptor with the membrane IgM molecules: CD79B chains are affected in 20% of the samples, whereas mutations in CD79A are less frequent. These mutations occur in the immunoreceptor tyrosine-based motif (ITAM)—a unique module linking antigen and Fc receptors to downstream tyrosine kinase signaling cascades, in most cases replacing the first tyrosine residue (Y196) within the cytoplasmic tail [43]. In around 9% of ABC-DLBCL (and a smaller subset of GCB-DLBCL), activation of BCR and NF-κB can be attributed to gain of function mutations within the coiled-coil domain of the *CARD11/CARMA1* gene [44] component of the CBM complex. Finally, genetic gains/amplifications of the *BIRC2* and *BIRC3* loci, encoding the cIAP1 and cIAP2 E3 ubiquitin ligases, respectively, were detected in as many as 16% of ABC-DLBCL, but rarely in GCB-DLBCL [45]. In the canonical pathway, cIAP1/2 are now recognized as integral components of CBM signaling. In ABC-DLBCL, elevated cIAP1/2 leads to K63 auto-ubiquitination and thus controls the recruitment of LUBAC and IKK to the CBM complex, thereby inducing IKK2 activation and increasing NF-κB levels.

The *MYD88* gene coding for the adapter of TLRs is mutated in 30% of ABC-DLBC. The L265P MYD88 mutation induces a spontaneous activation of the downstream IRAK complex, leading to engagement of the NF-κB pathway [46].

Bi-allelic truncating mutations or deletions have been observed in the *TNFAIP3*/A20 gene in one third of ABC-DLBCL cases and fewer GBC-DLBCL cases [47]. TNFAIP3/A20 possesses dual ubiquitin-editing functions. In particular, the N-terminal domain of TNFAIP3 is a deubiquitinating enzyme for K63-linked polyubiquitinated signaling mediators such as TRAF2/6 and RIP1 (Receptor Interacting Protein 1), while its C-terminal domain is a E3 ubiquitin ligase for K48-linked degradative polyubiquitination of the same substrates. TNFAIP3 has a specificity for particular polyubiquitinated substrates to regulate NF-κB activation in the TNF, IL-1β, and TLR pathways. TNFAIP3 mutations likely contribute to lymphomagenesis by inducing unregulated prolonged NF-κB responses. Some DLBCL patient biopsies show a deregulation of the glycolytic enzyme GAPDH that resulted in the activation of a NF-κB/HIF-1α (Hypoxia inducible factor 1) axis that could support lymphoma growth and vascularization through the induction of *VEGFR* [48].

The non-canonical NF-κB pathway is also aberrantly dysregulated in DLBCL, as 10–15% of GCB- and ABC-DLBCL carry genetic lesions in TRAF2 and TRAF3 associated with an activation of this pathway [49]. Recently, a high expression of the atypical nuclear IκBζ was observed in ABC-DLCBL [50]. The underlying molecular mechanisms for this phenomenon remain unclear. However, the increase in IκBζ is important for lymphoma survival by inducing p50–p52 homodimer target genes.

Hodgkin lymphoma (HL) is an unusual type of lymphoid malignancy: less than 1% of the cells in the affected lymph node correspond to malignant tumor cells—so-called Hodgkin and Reed/Stenberg cells (HRS). Constitutive activity of NF-κB was first identified 20 years ago in Hodgkin’s lymphoma-derived cell lines, as well as primary HRS cells [51,52]. Since then, numerous studies have been conducted and there is now evidence that both canonical and non-canonical pathways are enhanced in HRS cells. Indeed, RelB/p52 complexes can be found in HRS cell lines, indicating that the non-canonical pathway contributes to the neoplastic feature of HL [53]. This is further supported by the detection of NIK protein in HL cell lines and primary HRS cells [54].

HRS cells carry genetic lesions that lead to a gain or loss of function for several NF-κB pathway components. In about 50% of HL cases, the *REL* gene displays gains or amplifications. As a consequence, the levels of nuclear c-Rel are increased in HRS cells, likely contributing to constitutive NF-κB activation [55]. The two inhibitors IκBα and IκBε are also targets of mutations. Indeed, 10–20% of classical HL exhibit somatic inactivating mutations in the *IκBα* gene (*NFKBIA*), whereas mutations in the *IκBε* gene (*NFKBIE*) have been described in only one HL cell line and in one case of classical HL. This suggests that the genes encoding for NF-κB inhibitors may be considered as tumor suppressors in HL [56,57]. Some *BCL3* copy number gains leading to elevated expression of the proto-oncogene have been identified [58]. Bcl3 can enhance canonical NF-κB transcription and target gene expression after binding to p50 homodimers. Finally, recurrent deletions of the chromosomal region 6q23 involving *TNFAIP3/A20* have been found in HL cell lines and HRS cells, as well as inactivating *TNFAIP3* somatic mutations in several HL cell lines and in about 45% of classical Epstein-Barr virus (EBV)-negative HL cases [59].

Cell extrinsic mechanisms are also important contributors to NF-κB constitutive activation in HRS cells. Indeed, in a lymph node, the HRS malignant cells (less than 1% of the tumor mass) are surrounded by infiltrating T and B lymphocytes, monocytes, eosinophils, macrophages, and dendritic cells. These immune cells are attracted to the tumor site and activated by numerous cytokines/chemokines secreted by HRS cells, and in turn express various ligands that favor tumor survival. For example, CD40- and CD40L-expressing CD4^+^ T-cells are abundant in the HL microenvironment. These T cells surround HRS cells to form rosettes, suggesting a direct engagement of CD40 and thereby its activation [60]. Stimulation of CD40 induces NF-κB signaling in HRS cells and promotes the expression of its target genes coding for CD40 itself, the tandem molecules CD80 and CD86 involved in the priming of T-cells by dendritic cells, and of a set of anti-apoptotic proteins (Bfl1/A1, Bcl-xL, cIAP1) [61,62]. Another important player in the HRS-microenvironment crosstalk is RANK (receptor activator of NF-κB)—a member of the TNFR family capable of activating NF-κB. RANK and its ligand RANKL are expressed by HRS cells, suggesting an autocrine stimulation. In addition, RANKL is primarily expressed by activated T cells and dendritic cells. In HRS cell lines the activity of NF-κB is positively correlated with the level of RANK expression, suggesting that RANK/RANKL may contribute to the constitutive NF-κB signaling [63].

HRS cells express both CD30 and its receptor CD30L (CD153), suggesting another autocrine mechanism. However, the overexpression of CD30 leads to the recruitment and self-aggregation of TRAF2 and TRAF5, resulting in constitutive NF-κB activation and cytokine expression, independently of CD30L [64]. In HRS cell lines, TRAF1 is induced by CD30 signaling. This leads to TRAF-dependent processing of p100, increased p52 levels, and hence activation of the non-canonical NF-κB pathway [65]. Aberrant Notch activity is an also essential upstream regulator of non-canonical NF-κB activation in HRS cells, as NOTCH1 induces the processing of the *NFKB2* gene product p100 into its p52 active form and leads to an enhanced DNA binding activity of RelB/p52 heterodimers [66].

In about 30–40% of classical Hodgkin lymphoma in the Western world, HRS cells are latently infected by Epstein-Barr virus (EBV). The EBV^+^ HRS cells express three latent proteins: the EBV nuclear antigen (EBNA1) and the latent membrane proteins 1 and 2 (LMP1 and LMP2). The cytosolic domains of LMP1 carry two carboxyl terminal activating regions (CTAR) which bind TRAF proteins to mediate NF-κB activation, mimicking a constitutively-active CD40 receptor [67]. The important role of EBV in constitutive NF-κB activity is further supported by the observation that inactivating mutations in *TNFAIP3*/A20 gene and EBV positivity are largely mutually exclusive. In addition, *NFKBIA* mutations are mostly found in EBV-negative cells, suggesting that EBV infection can replace the role of some genetic lesions in HRS cells.

Multiple myeloma (MM) is an incurable plasma cell malignancy accounting for 13% of all hematological cancers. Disease progression involves clonal expansion of transformed plasma cells into tumors in the bone marrow. The heterogeneous tumor entity is characterized by long-lived plasmatic B-cells in the bone marrow. Elevated levels of NF-κB activity were found in relapsing MM, suggesting that NF-κB could be used as a prognostic marker as well as a target for therapy to prevent progression of the disease [68]. Constitutive activity of NF-κB has been found in multiple myeloma cell lines—especially in chemoresistant cell lines [69]. The same lab has also identified eight polymorphisms of the *IκBα* gene that were more frequently present in patients with MM [70]. The most frequent polymorphisms are located within exon 1, which encodes the N-terminal domain of IκBα containing the ser32/36, or within exon 6, encoding the PEST region. Gene expression analyses showed that 80% of myeloma biopsies display a high amount of *RelA* that correlates with enhanced expression of anti-apoptotic genes [71]. Keats et al. identified a promiscuous array of mutations that result in the constitutive activation of the non-canonical NF-κB pathway in approximately 20% of MM patients (inactivation of *TRAF2, TRAF3, CYLD, cIAP1/cIAP2*, and activation of *NFKB1*, *NFKB2*, *CD40*, *LTBR*, *TACI*, and *NIK*) [72]. Another clue for the implication of the non-canonical pathway is the strong nuclear accumulation of RelB in primary MM samples [73].

In MM, the crosstalk between malignant cells and the bone marrow stromal cells plays an important role in the pathogenesis of the disease, and largely affects the status of NF-κB. Indeed, NF-κB can be activated in MM cells by diverse bone marrow-derived cytokines and growth factors (e.g., BAFF/BAFF-R [74], APRIL/BCMA [75], CD40L/CD40 [76]) and by direct physical contact between MM cells and stromal cells [77].

## 3. Myeloid Malignancies

Acute myeloid leukemia (AML) represents a heterogeneous group of clonal stem cell malignancies arising from so-called leukemic stem cells (LSC). LSCs give rise to leukemic myeloid blasts arrested at different maturation steps. High proliferation rates of AML blasts are responsible for the invasion of bone marrow, and are associated with a fatal outcome.

The first observation of a constitutive NF-κB activity in AML was reported 16 years ago [78]. Indeed, Craig Jordan’s lab showed that primitive CD34^+^/CD38^−^/CD123^+^ AML cells aberrantly express active NF-κB, and that in vitro cell treatment with proteasome inhibitors is sufficient to induce rapid cell death. One year later, the first evidence of a dysregulation of IKK signaling in AML was published [79]. An increased level of IKK activity was observed in AML blasts derived from both BM and PB, associated with NF-κB activation. A more extensive activation was found in FAB (French-American-British) subtypes M4/M5, representing myelomonocytic/monocytic blasts compared to M1/M2.

Studies using the proteasome inhibitor (MG132) or the IKKβ inhibitor (AS602868) demonstrated that the pharmacological targeting of NF-κB induced cell death in AML cells both in vitro and in vivo, without affecting normal bone marrow CD34^+^CD38^−^ cells [78,80,81]. This led to the conclusion that AML cells are highly dependent on NF-κB signaling for their survival compared to normal bone marrow cells—a dependency that constitutes a therapeutic window for NF-κB targeting strategies.

Genetic alterations of *NF-κB* genes have not frequently been found in myeloid leukemia, suggesting that the origin of sustained NF-κB activation is different in AML than in other lymphoid malignancies. NF-κB activation is therefore a direct consequence of the chromosomal translocations/mutations that are characteristic of the different AML subtypes. The t(8;21) translocation is one of the most frequent ones found in AML, and produces the chimeric protein AML1-ETO. In normal hematopoietic cells, AML1 represses NF-κB signaling by interacting with the IKK complex, preventing their activation. AML1/ETO is unable to interfere with the activation of the IKK complex, leading to increased IKK activity, and consequently to aberrant NF-κB signaling [82].

Mutations in the *C/EBPα* (CCAAT/enhancer binding protein α) gene are detected in 10–15% of AML patients. C/EBPα and C/EBPα leucine zipper mutants could bind to the *p50* gene promoter, thereby elevating the expression of p50 protein. Moreover, p50 regulates *C/EBPα* expression in a positive feedback loop. It has also been shown that C/EBPα oncoproteins physically interact with p50 homodimers at the promoter site of the anti-apoptotic genes *BCL-2* and *C-FLIP*. As a consequence, C/EBPα proteins have the capacity to displace histone deacetylases (HDAC) from p50 homodimers to activate NF-κB target genes whose expression would otherwise be repressed [83,84].

Deletions involving the long arm of chromosome 5—del(5q)—are a common cytogenetic defect in high-risk MDS/AML (Myelodysplastic Syndrome/Acute Myeloid Leukemia). del(5q) MDS/AML employs an intrachromosomal gene network involving the loss of miR-146a and haploid overexpression of p62 via NF-κB to sustain a TRAF6/NF-κB signaling for cell survival and proliferation [85]. p62 facilitates K63-linked polyubiquitination of TRAF6, and consequently initiates NF-κB signaling.

The receptor tyrosine kinase Fms-like tyrosine kinase 3 (FLT3) is highly expressed in most patients with AML, and nearly 30% of them possess an internal tandem duplication (ITD) within the juxtamembrane domain that is associated with a poor outcome. The constitutive activation of FLT3-ITD is responsible for IKK activation through phosphorylation, thereby inducing the canonical NF-κB pathway [86]. However, FLT3-ITD also enhanced the phosphorylation and activation of TAK1 to activate the non-canonical NF-κB pathway [87]. TAK1-activated p52 binds to HDAC to repress transcription of DAPK1 (Death-Associated Protein Kinase 1)—an essential player in endoplasmic reticulum stress-induced apoptosis.

Since a proportional subset of AMLs express high levels of various cytokines, including the NF-κB activators TNFα, IL-1, and IL-6, autocrine signaling is also involved in constitutive NF-κB activation [88,89].

Disease progression is supported by cross-talk between AML cells and the bone marrow microenvironment. It has recently been shown that the engagement of VCAM-1 (vascular cell adhesion molecule 1) on stromal cells with VLA-4 (very late antigen 4) on AML cells induces reciprocal NF-κB activation in leukemia and stromal cells and triggers stroma-mediated chemoresistance [90].

Chronic myeloid leukemia (CML) is characterized by the chromosomal Bcr-Abl translocation, leading to the expression of the Bcr-Abl fusion oncoprotein that exhibits constitutive tyrosine kinase activity. Almost 20 years ago, NF-κB was described as an essential component of Bcr-Abl signaling [91,92]. Five years later, Kirchner et al. showed an NF-κB activation in primary CML samples from blast crisis patients for the first time [93]. Using a Bcr-Abl-transduced BaF3 cell line—an IL-3-dependent murine pro B-cell line—they showed that NF-κB activation is IKK-independent, but instead requires Ras activation. Nevertheless, other studies confirmed a contribution of IKKβ to the constitutive NF-κB activity observed in CML primary cells and cell lines. In these studies, two IKKβ inhibitors (PS-1145 [94] and AS602868 [95]) were able to promote apoptosis of CML primary cells. In particular, AS602868 was also efficient on CML cells expressing a T315I Bcr-Abl mutant that is resistant to all known tyrosine kinase inhibitors [95].

The microenvironment also plays a key role in the regulation of NF-κB signaling in CML. For instance, TNF-α supports the survival of CML stem/progenitor cells by promoting NF-κB/RelA pathway activity and expression of the interleukin 3 and granulocyte/macrophage-colony stimulating factor common β-chain receptor [96]. In CML, Bcr-Abl up-regulates TGF-β 1 expression, and released TGF-β 1 activates a PI3K/Akt/NF-κB/MMP9 signaling pathway from the stroma that subsequently results in the release of s-KitL and s-ICAM-1 (InterCellular Adhesion Molecule 1), ultimately enhancing the recruitment and mobilization of tumor stem cells to the peripheral circulation [97].

## 4. Conclusions

While a constitutive activation of NF-κB appears to be a frequent event in most (but not all) hematologic malignancies, it is amazing to observe that it results from a great variety of mechanisms and affects both the canonical and the non-canonical pathways. It is surprising that only rare mutations have been reported in the genes coding for the kinases that govern the two flavors of NF-κB activation—namely, IKKα and IKKβ for the classical pathway, and NIK and IKKα for the atypical one. For instance, it would have been expected that a mutated active IKKα that could activate both NF-κB pathways would have been found in many cases. Activating mutations have been reported for IKKβ in few (8/117) cases of MZL. It is possible that the active forms of these three kinases are too deleterious to be selected by cancer cells. In the opposite situation, inactivating mutations and deletions affecting the genes for IKKα, IKKβ, and NEMO have been described as generating several inherited diseases that display immune or developmental defects [98]. Constitutive NF-κB activation results from four main mechanisms: the presence of NF-κB activators in the leukemic environment, activating mutations in positive NF-κB regulators, invalidating mutations in NF-κB negative regulators, and finally, genetic defects resulting in amplifications of mutations of several NF-κB subunits. This diversity likely allows the stimulation of specific functions of NF-κB pathways without being too harmful. The specific consequences of activation of either the canonical or the non-canonical pathways on transformed cells are not presently known. One possibility to explore is that they may combine with different oncogenic events.

Interestingly, in several situations, it appears that the dysregulation of NF-κB follows two phases. In the first, the abnormal and sustained NF-κB activation results from influences from the leukemic microenvironment that secretes NF-κB activators or from chronic inflammation due to a bacterial infection and/or antigenic stimulation. Then, in a second phase, the occurrence of genetic events provides an autonomous activation of NF-κB that appears to lock on NF-κB activation to support further and complete transformation of the cell. A genetic activation of NF-κB is likely to be stronger and more sustained than upon chronic inflammation, which may result in a higher level of transcription of target genes, but could also modify the quality of the repertoire of activated genes.

The diversity in the defects that lead to abnormal NF-κB activation makes the possibility of finding a universal target difficult. Targeting IKKβ to block the canonical NF-κB pathway has been tempered by the fact that inhibition of IKKβ leads to enhanced IL1β expression and excessive inflammation [99]. Nevertheless, the different kinases [100] or proteases such as MALT1 [101] that participate in NF-κB activation are being evaluated as potential targets.
